# What are the functional outcomes of right hemisphere stroke patients with or without hemi-inattention complications? A critical narrative review and suggestions for further research

**DOI:** 10.3109/09638288.2015.1037865

**Published:** 2015-04-20

**Authors:** Maria Stella Stein, Cherry Kilbride, Frances Ann Reynolds

**Affiliations:** ^a^ Department of Clinical Sciences, Brunel University, London, UK

**Keywords:** Functional outcomes, hemi-inattention, modelling, neglect, right hemisphere, stroke

## Abstract

*Purpose*: There is widespread acceptance that patients demonstrating neglect/hemi-inattention (HI) following right hemisphere stroke (RHS) underachieve functionally compared to their counterparts without neglect. However, empirical evidence for this view needs examination. The purpose of this review is to critically appraise relevant studies that compared outcomes from RHS patients with/without hemi-attention and suggest more robust follow-up research. *Method*: Twelve studies published in 1995–2013 were critically reviewed. Two independent reviewers appraised design features including sample representation, assessment and data analysis methods. Strengths and limitations were highlighted. *Results*: Results were largely inconsistent. Considerable heterogeneity within patient groups and across studies complicated interpretation. Evidence suggested average group disparity in scores between patients with and without HI at discharge but the cause of functional disparity could not be attributed specifically to HI from the data and modelling results available. *Conclusion*: The relationship between HI status and functional recovery warrants further investigation in studies with stronger methodology to ensure rigour and robustness in the results. Pending further research, HI status should not be regarded as a key predictor of functional recovery or rehabilitation potential in patients with RHSs. This group should continue to receive appropriate therapeutic intervention aimed at maximising their functional recovery post-stroke.

**Implications for Rehabilitation**
- Findings from this review demonstrate a paucity of evidence to support the presence of hemi-inattention as a key predictor of functional recovery in patients with right hemisphere stroke; as such, practitioners should take this into consideration when planning rehabilitation programmes of their patients.
- In the initial months following right hemisphere stroke, there are wide-ranging differences in the rate and amount of functional recovery in patients, with and without hemi-inattention. Practitioners should not limit the aspirations of their patients based on the presence or absence of hemi-inattention.
- This review has identified a number of measurement limitations in commonly employed assessment tools for hemi-inattention and overall functional recovery. As such, practitioners should take the limitations of specific measures into account when interpreting the results contextually and with respect to their patients’ situation.

## Introduction

Hemi-inattention (HI), commonly referred to as “neglect”, is a complex, heterogeneous and disabling condition which acutely affects up to 80% of patients with right hemisphere stroke (RHS) dysfunction [1,2]. Despite considerable research and advances in the field, HI remains poorly defined as a condition *per se*. This is supported by the use of multiple descriptors (e.g. unilateral neglect, unilateral inattention) and taxonomies in the literature [3–5].

Clinically, HI is characterised by reduced attention and/or spatial awareness to details in the environment (commonly towards the left side of the body). HI can affect one or more functional domains (e.g. sensory-motor, visual-spatial) [6,7] and often co-exists with anosognosia and depression [8,9]. HI has been regarded as responsible for delayed and challenging rehabilitation, reduced safety awareness, poor functional outcomes, increase in dependency levels and risk of institution care [10–12].

Historically, findings from published studies have reported disparity in functional ability scores; with patient groups affected by HI (HI+) underachieving compared to those without (HI−) [11,13,14]. Traditionally the cause of this disparity has been largely attributed to the presence of HI, although findings from predictive models have been conflicting and inconclusive [10,13,15–17]. This has led to considerable confusion and uncertainty about the clinical importance and significance of differences thought to be associated with HI [9,18–20]. The paucity of relevant evidenced-based reviews has not helped to clarify the predictive role of HI or to promote good rehabilitation practice.

The last systematic review was undertaken by Jehkonen et al. [21]. The authors focused on the methodological quality of 26 studies published in 1996–2005, which evaluated the impact of Neglect on functional ability in predominantly generic stroke patient samples with mixed lesion sites. Jehkonen et al. [21] highlighted as an issue considerable differences in patient samples and inconsistencies in results but nonetheless concluded that HI had a significant negative impact on functional outcome, either as an independent predictive factor or in the presence of other variables. Their findings corroborated those of earlier reviews [2,22] which were not specifically focused on the relationship between HI and functional ability. Jehkonen et al. [21] recommended further research on homogeneous patient groups with respect to right/left hemispheric lesions to improve consistency in the results.

Given the paucity of research in this area, an in-depth evidence-based critical review of relevant studies is offered here with a different approach to that taken by Jehkonen et al. [21]. However, considering the extent of methodological differences between studies [21], a narrative review was appropriate. This enabled the inclusion of sufficient, relevant contemporary studies which would have otherwise been excluded by the more stringent selection criteria of a pure systematic review. Narrative reviews “lay out the most recent and best knowledge of various aspects of a problem” [23, p. 427], and are considered appropriate when a diversity of research methods are used in the studies considered as relevant (rather than focusing only on randomised controlled trials), where studies have used different outcome measures and/or non-equivalent samples [24] and when studies are of relatively poor methodological quality [25].

The current review examined traditional claims made by previous studies and reviews [21,22] about the negative impact of HI on function; more specifically the strength of the relationship between HI status and functional recovery following RHS. Another aim was to estimate the magnitude of functional differences between HI± patient groups. The current review extended the work carried out by Jehkonen et al. and used a more rigorous and systematic approach to the selection of studies and the review process. Consequently it included fewer (*n* = 12) but more homogenous studies with RHS patient samples. Theoretically similarly designed studies tend to be more comparable than heterogeneous stroke studies.

Both the discrepancy in HI± patient scores and the relationship between HI and functional recovery are of interest to rehabilitation professionals. Together with other indicators (e.g. stroke severity) they may be used to predict likely change in function with time since stroke. This knowledge can guide rehabilitation decisions, e.g. as to which patients are suitable for early supported home discharge schemes. Currently there is an urgent need for reliable predictors and indicators to support the transfer of in-patient rehabilitation services to appropriate stroke survivors in the community. The final aim was to formulate more robust research strategies based on the limitations of studies to date.

## Method

A literature search was conducted from 1995 to February 2015 of the databases MEDLINE, AMED, CINAHL, PsycINFO and COCHRANE using several descriptors of *neglect* subtypes in the literature including *HI*, spatial, visual, unilateral, personal, extra-personal, motor, sensory, hemi and *representational.* The words; stroke, CVA, functional* and activities of daily living (ADL) were added to the final search so that studies focused on specific functional activities were included. Children or young adults (≤18 years) and non-human samples were excluded.

The search yielded three Cochrane reviews and 195 publications; AMED (70), CINAHL (86), MEDLINE (102) and PsycINFO (57). In line with the aims of the review, and supported by recommendations from Jehkonen et al. [21], only studies that compared the homogeneous patient groups with respect to hemispheric lesion site (RHS) and presenting comparisons of patients with or without HI were selected (including intervention designs); all other studies with the heterogeneous patient samples and no HI group comparison were excluded. In addition, functional ability had to be quantifiably measured so that the HI± group differences in scores could be calculated. Two reviewers read the abstracts and, when in doubt, the publication to determine relevance. This process led to the selection of 12 international studies.

The following information (source, aims, design type, demographic data, assessment tools, data analysis method, results and findings) were extracted from each study and are summarised in Table 1. The critical evaluation process was guided by a checklist described in Appendix 1. Importantly, it focused on the extent of representation of the RHS patient sample with respect to stroke and HI severity levels, time to baseline assessment and follow-up observations, the type of data collected and appropriateness of assessment tools, extent of statistical data analysis undertaken including modelling specifications and, where appropriate, the extent of adjustment undertaken for established confounding factors (e.g. age, stroke severity, time since stroke) and handling of missing data. Each study’s strengths and limitations were identified as part of the review process, these are summarised in Table 1; also included is the authors’ assessment of the methodological quality of each study. This was graded (A–D) according to the Grading of Recommendations Assessment, Development and Evaluation (GRADE) [26].

**Table 1.** Critical evaluation of reviewed studies (abbreviations are defined in Appendix 2).

Source	Aims and design	Assessment/tools	Data analysis	Results/findings	Study strengths	Study limitations
Kalra et al. (1997) (UK) [15] GRADE C	Aim – RCT to determine whether poor outcome in patients with visual neglect (VN) was due to greater stroke severity or non-specialist management Setting – Acute, stroke unit Sample (47 HI+, 99 HI−) Mean age 77 (SD = 8) Time to 1st obs. 1–2 weeks post-stroke onset Follow-up at discharge Before and after controlled intervention (conventional versus spatio-motor cueing and early emphasis on restoration of function)	VN assessed by Line bisection supplemented by functional observation at admission 1^a^ Outcome BI (scale 0–20) and Thumb finding test 2nd Outcome Mortality Discharge-destination LOS Therapy intensity	Median statistic Chi squared test, Mann–Whitney *U, t*-test Multiple linear regression (*n* = 146), DV = BI at admission Modelled IVs Age, gender, muscle power, balance, proprioception, cognition, pre-stroke ADL status, HI level	Patients with or without visual neglect (VN) had similar destination, slightly lower median BI scores at admission and discharge (4 versus 5 and 16 versus 14) resp. Greater LOS/days (64 HI+ versus 36 HI−) and therapy input/h PT (30 HI+ versus 19 HI−) and OT (18 HI+ versus 10 HI−) HI negatively associated with admission BI [β = −0.17, *p* = 0.011, R2 = 0.16] All other IV’s not associated with DV	Confirmed stroke Clear selection criteria Validated ADL assessment Statistically modelled variety of factors associated with ADL besides HI Reported attrition due to death (*n* = 3 extended stroke, 1 pulmonary embolus, 1 myocardial infarction) Intention to treat analysis Corrected for small sample size	Wrongly labelled as RCT Recruited only patients with Partial Anterior Circulation Infarct of moderate stroke severity with potential for rehabilitation Line bisection does not distinguish between visual neglect and other sub-types BI version excluded psycho-social dysfunction & cognitive measure No community follow-up Different patient LOS so exposure to therapy uncontrolled Did not model outcome data at discharge No sensitivity analysis
Ring et al. (1997) [28] (Israel) GRADE C	Aim – To measure function and determine gain between admission and discharge Design – Prospective comparative Setting – Acute General Rehabilitation facility Sample (28 HI+, 56 HI−) Mean age 60.8 Time to 1st observation was 29 days (±17) Follow-up at discharge	BIT at admission to detect “neglect” 1^a^ Outcome FIM 2nd Outcomes LOTCA Type and site of lesion LOS Discharge destination	*t*-test Chi square test Repeated measures ANOVA Multiple linear regression with FIM gain (DV) Modelled IV’s LOS, admission FIM, age, gender, risk factors (not clear which)	FIM admission score, LOS and age predicted functional gain [β = −0.034, 0.13, 0.49, *p* = 0.011, 0.03, 0.05] resp. 24/28 patients with HI discharged home after considerably longer period of rehab and LOS/days (137 HI+ versus 102 HI− days) Total FIM gain HI+ 33 versus HI− 21 units	Confirmed stroke by CT scan Validated functional ability scale and test battery for detection of HI Statistically adjusted for age and gender Clear distinction between RHS and LHS, lesion site and type Reported attrition due to death (*n* = 1)	Selection criteria not clear what behavioural conditions were excluded Variable obs. time-point No community follow-up Not adjusted for differences in stroke severity or time post-stroke No sensitivity analysis No data on cognitive function from LOTCA published
Paolucci et al. (2001) [16] (Italy) GRADE C	Aim – Assess influence of unilateral spatial neglect (USN) on rehabilitation outcome Matched by Age (69 ± 10) and stroke onset admission time (38 ± 17 days) Setting – Acute, in-patient rehabilitation hospital Sample – (89 HI+, 89 HI−) Time to 1st observation (38 ± 17 days) Follow-up at discharge Intervention; special training in visual scanning, reading and copying script, line drawings, dot matrix and description of scene 5h/week for 8 weeks	USN detection – Letter cancellation, line bisection, sentence reading and Wundt–Jastrow area illusion test at admission 1^a^ Outcome BI (0–100) 2nd outcome LOS Rate of gain and amount of progress Other RMI CNS Hamilton Depression Rating scale	Eight multiple linear regression (forward stepwise) Six logistic regressions Five DV’s, CNS, BI, RMI, LOS, rate of gain and amount of progress Modelled IV’s Admission CNS, gender, type of lesion, hypertension, diabetes, heart disease, unilateral spatial neglect, depression, epileptic seizures post-stroke, family support, education level, discharge destination	USN was a negative prognostic factor. USN patient group had low ADL and mobility outcomes at discharge (∼50% less mean scores) HI+ had longer LOS/days (117 ± 61 versus 81 ± 38), ↑rate of discharge to institution (18% versus 5%), ↑ discharge continence rates (21% versus 5%) USN, stroke severity, heart disease and type of lesion appear to be important explanatory variables in the acute phase (∼3 months)	Confirmed stroke (CT scan) Validated tools BI supplemented by data from RMI Screened for depression and neurological severity Reported attrition, (9% HI−, 6.7% HI+) Modelled broader range of factors e.g. psych-social factors and comorbidity Adjusted for stroke severity in some models	Probable patient overlap with earlier sample (Paolucci et al. [27]) Probably excluded severe stroke included (mean CNS = 7) Highly variable T0 observations Complicated paper to follow due to large number of factors and combinations modelled Did not measure cognition which is strongly associated with USN (neglect) Not adjusted for or modelled age which is associated with USN High variability in LOS and exposure to in-patient care likely source of bias No information on handling of missing data
Buxbaum et al. (2004) [11] (Italy and USA) GRADE D	Aim – Assess occurrence of subtypes and related deficits in RHS Design – cross-section, retro and prospective data Setting – Acute and community Sample – 623 RHS recruited from four rehab hospitals in Philadelphia and two in Italy. 268 met selection criteria 166 consented; 86 had acute and 80 chronic lesions, (88 HI+, 78 HI−) Mean age – Acute 66, range (37–89) chronic 67, range (33–88) Time to 1st and only observation – Acute (5–41) and chronic (94–1272) days	Personal and Peri-personal Bells test and 4 Behavioural Inattention (BIT) sub-tests (letter cancellation, picture scan, menu reading and line bisection) Motor and perceptual neglect measured by response latencies in two stimulus and response tasks Motor and Sensory exam visual fields and extinction by means of confrontation method Sustained and divided auditory attention Test (SART) Anosognosia 5 questions adapted from Cutting’s questionnaire 1^a^ Outcome FIM Family Burden Scale	Chi square test Mann–Whitney *U* test Correlation tests Repeated measures ANOVA Regression analyses	Neglect severity significantly explained FIM scores and carer burden but not lesion size Similar rate of gain in HI± but lower FIM scores in HI+ (estimates not reported in paper) Acute patient lesions were not restricted to cortical areas Variation in associated deficits but higher frequencies in HI+ Variation in occurrence of HI sub-types	Attempted to document frequency of various HI subtypes and related deficits Included burden of care assessment Acknowledged significant limitations in sensitivity and specificity of tests used to identify neglect sub-types and anosognosia Also acknowledged lack of statistical adjustment for multiple tests	Significant heterogeneity in sample and variation in time to 1st observation complicate interpretation of results Recruited patients deemed to benefit from rehabilitation, i.e. Excluded severe attention and cognitive deficits, previous stroke or neurological disorder and dementia Combined analysis of patients from different culture and health care systems – can be strength but also weakness Inter-rater reliability not performed FIM mean scores not directly reported
Gillen et al. (2005) [12] (USA) GRADE D	Aim – Examine the relationship between left unilateral spatial neglect (USN) and rehabilitation outcomes in RHS patients Design - Retrospective Setting – Acute in-patient rehabilitation hospital Sample – (50 HI+ 125 HI−) Mean age 72 (SD = 11.0) Time to 1st observation was 15 ± 10 days Follow-up observation at discharge	“USN” assessed by Letter cancellation test (LCT) at admission 1^a^ Outcome FIM Other Cognistat at admission Geriatric Depression Scale (GDS) at admission LOS	Univariate correlation Multivariate regression analyses (*n* = 98) FIM discharge scores (DV) regressed on FIM admission and USN	Longer mean LOS in HI+ 31 versus 25 in HI− HI+ progressed at slower rate. Mean admission FIM score 50 (SD = 16) versus 69 in HI− (SD = 16) Greater cognitive impairment in HI+ (p < 0.001), higher GDS scores and depression levels (p < 0.01) “USN” predicted social-cognitive domain (β = −0.29, p < 0.001)	Included depression and cognitive function Used validated measures Modelled rate of progress (change in FIM score/LOS)	106/281 eligible patients excluded due to poor visual acuity. Perceptual deficits and difficulty completing LCT at 1st observation Depression assessed probably too early when patients are likely to be depressed due to stroke event No FIM or cognitive discharge score reported.
Odell et al. (2005) [29] (USA) GRADE D	Aim – To document selected functional outcomes at the termination of in-patient treatment Design – Retrospective Setting – Acute in-patient rehabilitation hospital Sample – (60 HI+ 41 HI−) Mean age 70 years Range (40–99) Time to 1st observation not known Follow-up observation at discharge	No formal assessment of HI (relied on mention of condition in medical records) 1^a^ Outcome FIM scores at admission and discharge 2nd Outcome Amount and efficiency of gain, LOS Discharge placement	Mann–Whitney *U* test Regression analysis Modelled IV’s 12 predictor variables made up of initial motor score, cognitive items plus age, gender, previous neurological episodes, no. of comorbidities, lesion site and presence/absence of HI	Admission, discharge FIM median HI+ (57 and 88), HI− (66 and 104); similar gains in motor ∼24 units, cognitive domains HI+ (3.5), HI− (2). 1 unit gain in FIM cognitive scores by in HI± groups When modelled, functional outcome was predicted by age, memory, problem solving and motor function Mean LOS, HI± 29 versus 22 (3–75) days; >75% home discharge Therapy sessions HI± 61 versus 27 (range 1–194)	Transformed data by means of Rasch method to increase accuracy of estimates Adjusted for variation in age Recorded number of comorbidities and therapy sessions Categorised descriptive statistics by age range (40–92); younger age group were less impaired and made highest gains overall	Highly selective criteria, i.e. included only patients referred to speech therapy (reduces generalisation of findings) Stroke severity not known No formal assessment of HI Variable follow-up observation point Limitations of retrospective studies, e.g. reliability and accuracy of data cannot be checked, consistency of assessment methods and data collection cannot be guaranteed Missing data not reported
Di Monaco et al. (2011) (Italy) [30] GRADE C	Aim – To investigate the relationship between severity of unilateral spatial neglect (USN) and functional recovery in ADL after a RHS Design – Prospective Setting – Acute in-patient, physical medicine and rehabilitation hospital Sample – (54 HI+, 53 HI−) Mean age 70 (range 63–80) Time to 1st observation was 23 days post-stroke onset Follow-up observation 80 days post-stroke onset	Detection of USN – BIT at admission only and Diller’s test (cancellation task) 1^a^ Outcome Admission and discharge FIM scores Other BI prior stroke by anamnesis Mini-Mental (MMSE) LOS	Data analysis on 107/131 Bivariate correlation FIM × BIT scores Mann–Whitney *U* test for group differences Chi square test Three multiple regressions Three DV’s = discharge FIM, FIM efficiency and effectiveness Modelled IV’s Age, MMSE score, time to 1st observation, gender, education, BI , FIM admission and discharge	Admission, discharge FIM median HI+ (45 and 91), HI− (55 and 110) but >30 units of variation within each group at all times MMSE median group score (HI+ 24, HI− 27). FIM admission best predicted FIM discharge score Model explained 49% of variance in DV; of these “USN” explained 5%; FIM 44% High variability in and LOS (37–72 days)	Reported missing data (*n* = 5) Statistically adjusted for age, gender, education level, time to 1st observation and FIM admission Transformed FIM scores to ∼ normal distribution Recognised limitations of the study, i.e. assessing limited no. factors associated with HI and function and limitations of BIT in distinguishing between sensory motor HI, visual-spatial and motor Modelled education level	Excluded 19 with severe stroke No intention to treat analysis – possible bias towards milder stroke severity (MMSE scores at admission indicate mild cognitive impairment) FIM cognitive score not provided to compare with MMSE No adjustment for stroke severity or carer status Different patient exposure to in-patient care likely source of bias
Timbeck et al. (2013) [32] (Canada) GRADE D	Aim – Evaluate effect of visuo-spatial neglect (VSN) on functional outcome and discharge destination in RHS Design – Prospective Setting – Stroke rehabilitation programme Sample - (6 HI+, 10 HI−) Mean age 76 (SD = 10) Time to 1st observation was 7 days from admission to rehabilitation Follow-up observation prior to discharge	VSN detected by BIT 1^a^ Outcome FIM Other MMSE Berg balance scale (BBS) CMSA LOS	MANOVA to compare between VSN± patients DV – age, time to 1st observation, LOS. MMSE, admission–discharge FIM, BBS and CMSA Independent *t*-tests for univariate analyses and Fisher’s exact for categorical variables	VSN+ (*n* = 6) tended towards supported living FIM admission–discharge score; HI+ 60 and 73, HI− 86 and 102 units High SD in both groups at all FIM observations ∼20 admission, 28 discharge LOS average VSN+ 48, VSN− 38 days Differences in BBS within groups (SD = 16), between groups; HI+ scored 12 and 22 versus 28 and 41 BBS units in HI− at admission and discharge resp	Included balance measure Supplemented motor activity on the FIM scale with another impairment measure Evaluated multivariate effect by Pillai’s trace (ensure robustness against non-normal distributions and heterogeneity of variance particularly with small samples and groups) Acknowledged significant study limitations	Very small sample unlikely to be fully representative of RHS has implications for study power and validity of results Tight selection criteria excluded patients with chronic co-morbidity (not clear what), English as 2nd language and cognitive impairment – has implication for generalisation of results Not accounted for changes due to spontaneous recovery effects occurring in average 28 days (SD 19.23) delay in starting rehabilitation programme. This has implications for findings and conclusions based on results No adjustment for multiple testing especially on a small sample
Paolucci et al. (1996) [27] (Italy) GRADE D	Aim – to test whether specific neglect training improved hemi-spatial neglect (HSN) and functional outcome Design – Prospective, cross over design for HSN+ (divided into two groups) + HSN- (3rd group) Setting – Community rehabilitation facility Sample *n* = 59 RHS (23 HI+, 36 HI−) Mean age 65 (SD = 13) Time to 1st observation was 2–6 months post-stroke onset Follow-up at 2 and 4 months whilst in rehabilitation Intervention – 40 h of visual scanning, auditory cueing, reading, copying, line drawing, picture description	“HSN” assessed once at admission to rehabilitation facility by Letter cancellation, line bisection, sentence reading and Wundt–Jastrow area illusion test at admission 1^a^ Outcome BI (0 to 100) Other RMI CNS Lesion size	Three ANOVA’s to estimate differences between three groups in BI, RMI and CNS scores at follow-up (2 and 4 months) Four ANOVA’s for differences in USN tests One ANOVA difference in lesion size by group (*n* = 3)	Specific USN training improved functional ability of USN+ group but gains not maintained by end of study Similar magnitude of difference between USN± patients in mean functional ability and mean RMI (1st, 2nd and 3rd observatio*n* = 20%, 30% and 30%, respectively) No group difference in lesion size	Screened for stroke severity but data not reported Validated measures Test-battery used to assess HI but not standardised Used RMI to supplement information on functional ability not provided by BI scale, e.g. walking outside house Community follow-up	No radiologic confirmation of stroke Excluded patients over 78, multiple lesions, haemorrhage or chronic CNS pathologies Crossover intervention for USN+ group sizes were small (*n* = 11 and 12) Stroke severity not known No fixed assessment time-points Not adjusted for multiple testing No statistical adjustment for confounding factors Attrition not reported
Katz et al. (1999) [10] (Israel) GRADE D	Aim – To evaluate impact of unilateral spatial neglect (USN) on functional outcome in long term Design – Prospective, longitudinal Setting – Acute, General Rehabilitation Hospital Sample – (19 HI+, 21 HI−) Mean age 57 (SD = 10) Time to 1st observation was ∼30 days Follow-up at discharge, 6/12 after discharge, up to 1 year post-stroke onset No intervention but USN+ patients received special attention and care for USN	USN detected by BIT at admission and discharge 1^a^ Outcome FIM Other LOTCA at admission and discharge RKE at discharge LOS	*t*-test Chi squared test Repeated measures ANOVA Multiple linear- regression – FIM (DV) Modelled IV’s (stepwise entry method) BIT score, sitting balance, thinking operations (not defined) and tactile sensation	USN was major predictor of functional outcome from admission to follow-up Despite special attention given to USN+ group, they had higher disability levels, slower improvement rate Most progress occurred within the in-patient facility Longer LOS/days for USN+ (119 ± 49) versus (78 ± 52) for USN− 39/40 patients were discharged home, one patient with USN discharged to nursing home USN+ needed high levels of support at home compared to USN− USN could be predicted from pen and paper tests alone (no advantage in giving functional sub-section)	Confirmed stroke by CT scan Long term follow-up 2/4 fixed observation points Modelled also cognitive, IADL score, tactile factors, sitting balance Reported therapy time 45–60 min of OT and PT/patient Tracked recovery of function up to a year post-onset	Small sample size, possibly underpowered for regression analysis (increased risk of type 1 error) Excluded severe stroke and psychiatric disorders not clear which, restricted inclusion to 1st stroke only with no comorbidities Inconsistent assessment protocol (BIT and LOTCA not repeated at follow-up) to assess recovery No attrition reported Observations from same patients not independent – invalidates regression assumption No statistical adjustment of confounding factors FIM is a multi-disciplinary tool (not clear how this was completed in the community?
Cherney et al. (2001) [14] (USA) GRADE D	Aim – To evaluate relationships between unilateral spatial neglect (USN) and cognitive-communicative functional outcomes in RHS Design – Prospective, repeated measures Setting – Acute rehabilitation facility Sample (36 HI+, 16 HI−) Mean age – 66 (SD = 14.0) Time to 1st observation at facility was 33 ± 68 days after stroke Follow-up at discharge and 3 months post-discharge	USN detected by (BIT) at admission 1^a^ Outcome FIM Other RIC-FAS LOS	ANOVA Mann–Whitney *U* test Pearson’s coefficient of correlation	Statistically significant differences were found in overall FIM and motor sub-score but not cognitive score. USN+ patients scored 10 FIM units (8%) less at each observation point High correlation between pen and paper tests and behavioural section on BIT (*r* = 0.89) Moderate correlation (*r* = 0.51) between FIM and BIT scores at 1st and 2nd observation points which weakened by 3rd (*r* = 0.36) LOS/days for USN+ versus USN− (38 ± 9 versus 31 ± 10). No impact of USN severity on LOS reported	Evaluated cognitive function and communication (not previously included) Reported attrition (*n* = 4) due to incomplete documentation at discharge and (*n* = 12) lost to 3 month follow-up	Small sample size for sub-group analysis by USN severity Highly variable time to 1st observation (source of bias) Stroke severity not known No fixed observation point – limits comparison of results No intention to treat analysis FIM scores at 3 month follow-up obtained by telephone interview – reliability of data?
Stein et al. (2009) [31] (UK) GRADE D	Aim – To compare and evaluate basic functional mobility in patients with and without visual neglect (VN) Design – Prospective, repeated measures Setting – Acute inpatient (stroke unit) and community rehabilitation Sample – (14 HI+, 14 HI−) Mean age 76 (SD = 11) Time to 1st observation was 7–28 days post-stroke onset Follow-up observation at discharge and 4 weeks post-discharge	VN detected by BIT 1^a^ Outcome BI (0–20) Other Elderly mobility scale (EMI) Middlesex elderly assessment of mental status (MEAMS) Postural assessment scale for stroke (PASS), LOS Discharge destination Continence status Carer status	Mann–Whitney *U* test Kruskal–Wallis Wilcoxon matched pairs Bonferroni correction for multiple testing	Mean LOS/days was 79 and 52 for HI±, respectively Seven VN+ discharged home versus 12 VN−. VN+ increased risk for institution discharge. Mean difference of 7 BI units (35%) at discharge (*p* = 0.013) Patients with mild VN and independent mobility tended to be discharged home Relationship between carer presence and discharge destination was not clear	Included community follow-up Included range of severity of VN levels Included separate measure of posture relevant to functional mobility Included data on discharge destination and continence status Reported number of deaths (*n* = 3) and outliers (*n* = 4) Corrected for multiple testing to minimise type 1 error	BIT, MEAMS, BI were not assessed post-discharge, therefore unable to track change especially in functional mobility Possibility that differences observed between patients could be due to type 1 and II errors largely due to small sample size No correlation statistics to study association of factors with functional mobility No fixed observation points limits comparison to other studies

On the GRADE scale; A is high and assigned to well-performed Randomised controlled trials (RCTs) and observational studies with consistent results and/or strong effects. B is moderate – serious flaws in the design in which the estimated effect is likely to be considerably different than the true effect. C is low – studies with serious limitations in which the true effect is likely to be very different than the estimated, e.g. failure to include relevant confounding factors. D is very low – as in C but any estimated effect is very uncertain and highly unlikely to reflect the true effect.

## Results

### Population studied and demographics

Geographically, studies were undertaken in Canada (1), Italy (3), Israel (2), UK (2), USA (3), USA & Italy (1) with local RHS populations. Two of the studies probably used the same population [16,27].

Age and gender were described consistently; study [11] made reference to educational background and family burden. The age range varied from 57 (SD 10) [10] to 60–69 [11,14,16,27,28] and 70–76 years [12,15,29–32]. In addition, there was considerable variation in age within specific studies, e.g. 33–88 years [11] and 40–99 years [29]. Gender tended to be equally distributed. Morbidity was documented in studies [16,29]; stroke was associated with hypertension, diabetes and heart disease. Stroke severity was not always made clear. It was reported as moderate in two studies [15,16], whilst study [30] indicated that patients with severe stroke were excluded. Two studies [11,14] recruited only patients with (perceived) good rehabilitation potential. Stroke severity was unreported in seven studies [10,12,27,28,29,31,32].

### Definition of function and “Neglect/HI” syndrome

Conceptually, functional ability/outcome was rarely defined but inferred from ADL measurement scales, mainly the Barthel Index (BI) [33] and Functional Instrumental Measure (FIM) [34]. “Neglect/HI” tended to be traditionally defined as a failure to orient, report or respond to stimuli located on the opposite side to the site of the brain lesion which cannot be explained by either primary sensory or motor deficits [35]. Different studies referred to HI sub-types interchangeably, e.g. visual neglect [15], unilateral spatial neglect [12] but effectively measured the same condition because the measurements used cannot differentiate between sub-types of neglect (e.g. visual, spatial and unilateral) [3,4,36] but provide an overall measure of the degree or profundity of the condition.

### Research settings

Research settings were insufficiently described to allow clear comparison between countries, e.g. termed as a rehabilitation facility or hospital in Israel and a stroke unit in England. They tended to be either acute in-patient hospital and/or community rehabilitation facilities which would suggest research on samples assessed at varying intervals after stroke onset.

### Design

Nine studies [10,14–16,27,28,30–32] employed a prospective design, two studies [12,29] employed a retrospective design and one study [11] employed both. Study [11] employed a cross-sectional design; most studies [5,12,16,28–30,32] employed a serial design characterised by variable baseline (T0) measures and one follow-up at discharge. Four studies [10,14,27,31] included up to three follow-up observations. The longest follow-up period was one year since stroke [10]. All other follow-ups were not fixed in time but varied relative to the discharge point.

### Selection criteria

Inclusion criteria tended to be vague; 10/12 studies [10–12,15,16,27–29,31,32] included only patients with “good rehabilitation potential” which was not clearly defined. However, by inference it would appear that severely cognitively impaired patients and those with common (age-related) morbidities (e.g. cardio-pulmonary) were automatically excluded early on from most of the studies.

### Confirmation of stroke

Stroke was reportedly confirmed by a neurologist in all the studies and by radiological means in 42% [10,14,16,28,31]. Stroke severity was measured in three studies [15,16,27] but the scale score was only reported once in [16] (using the Canadian Neurological Scale – CNS = 7). Aetiologically, infarct was predominant but the majority of studies also included haemorrhage.

### Time to first (1st) observation

Baseline measures were taken at variable (non-comparable) times across the studies, making direct comparison difficult. Time to 1st observation was not reported in study [29] and unclear in [32]. In hospital settings, initial measurement varied from 7 to 15 days since stroke in studies [12,15], up to 30 days [10,28,30,31], up to 40 days [14,16] but occurred after 2–6 months in community rehabilitation facilities [11,27].

### Sample size

Sample size and composition varied considerably; from 16 participants [32] to 178 [16]. Six studies [11,12,15,16,29,30] had more than 100 participants; three studies [14,16,28] reported between 50 and 100; three studies [10,31,32] had less than 50 participants. The proportion of HI+ to HI− patients also varied; 7/12 studies had less than 50% HI+ in the sample, the smallest being 19% [10] and largest 60% [29] (the latter being therefore more adequately powered to statistically detect differences between the HI± groups).

### Attrition rates

Attrition rates of 1%, 16%, 7.8% and 11% were reported [33,14,16,31], respectively. Reasons for attrition were due to a combination of factors (incomplete documentation at discharge, loss to community follow-up and mortality).

### Assessment of Neglect/HI

In regard to HI, both diagnostic tools and frequency of assessment varied; most studies [11,12,14–16,27–30] assessed only at baseline (which differed in time across studies), whilst three studies [10,31,32] assessed patients at admission and discharge (which also differed in time since stroke). In half of the studies [10,14,28,30–32], HI was identified and assessed by a validated test battery – the Behaviour Inattention Test (BIT) [37]. Single letter cancellation and line-bisection (pen and paper tasks) were used in two studies [12,15], respectively, whilst three used various standardised Neglect-specific tests [11,16,27]. Study [29] relied on mention of Neglect in the medical documents.

### Other assessments

Functional ability was assessed by the FIM in all but four studies [15,16,27,31] which used different versions of the BI. Most studies measured or recorded additional factors which ranged from length of stay (LOS), discharge destination outcome, continence status, aspects of cognitive-motor function including perception, muscle strength, balance and tactile sensation. Validated measurement scales were generally used for these purposes.

### Statistical data analysis

Data tended to be summarised by group (HI±) scores. Median or mean statistics were frequently reported with standard deviation (SD), and inter-quartiles to a lesser extent. Data distribution was rarely described but inferred from the summary statistic used. Rasch data transformation was undertaken by two studies [29,30] in an attempt to “normalise” a skewed data distribution. Estimation and management of missing data were not specifically reported, with one exception [30].

### Type of data analysis

The type and extent of data analysis varied substantially. For clarity, only general tendencies are described in this section; for specific details refer to Table 1.

The majority of studies carried out preliminary tests for uni/bivariate associations (e.g. neglect × functional ability) and/or group (HI±) score comparison, e.g. [11,12,30]. The correlation coefficients used were Spearman’s rho or Pearson’s *r*, whereas *t*-test, Mann–Whitney *U* and Chi square test were frequently used for group comparisons. In order to minimise type I error (i.e. a false statistically significant result), one study [31] adjusted for multiple testing of the same participants over time by means of Bonferroni correction. Adjustment for small sample size was reported in two studies [15,32] but the adjustment method (Pillai’s trace) was only described once in study [32].

Eight studies [10–12,15,16,28–30] used regression methods to evaluate various relationships between predictor or explanatory variables (IV’s) with one or more dependent variables (DVs), including functional ability. However, the type of model used was not clearly identified (predictive versus associative model). Therefore, it was difficult to assess the suitability of the models employed for the purpose of answering the question posed. For example, Paolucci et al’s study [16] modelled the impact on later function of a large number and combination of IV’s (admission stroke severity score, gender, type of lesion, hypertension, diabetes, heart disease, unilateral spatial neglect, depression, epileptic seizures post-stroke, family support, education level, discharge destination in various combinations) but the extent of adjustment for confounding factors was variable – stroke severity was inconsistently adjusted for and no adjustment for differences in age was undertaken. Therefore, it was difficult to tease out specific relationships and infer cause from complex regression models. Differences in age, gender and duration of in-patient stay were adjusted in some models evaluated by studies [11,16,30]. Furthermore, the rationale behind the choice, order of entry and measurement level (continuous/categorical) of IV’s was rarely stated (e.g. study [10] used stepwise methods, whereas study [16] used forward stepwise), which complicated understanding and interpretation of the results.

Three out of four studies with more than one follow-up point evaluated change in functional ability over time by means of ANOVA’s and/or multiple regression analysis [10,14,27]; study [10] specified repeated measures ANOVA. However, both methods have considerable limitations which potentially impact on study power and accuracy of results, especially in serial models with more than one follow-up and several repeated measures on the same individual. To this end, ANOVA requires complete data sets, which is problematic in stroke research due to the likelihood of missing data in the long-term. Ordinary single or multivariate regression analysis does not take into account correlation generated by multiple responses from the same individual on the same assessment measure/s (this violates the statistical assumption of independent observations in regression analysis [38,39]). Consequently, both the validity and accuracy of ordinary regression results are threatened including any inferences based on the results.

## Main results and findings

A substantial number of findings were reported across studies, only those pertaining to functional outcome and HI are summarised in this section (refer to Table 1 for details by study).

### Disparity between the HI± group scores

All the studies found statistically significant disparities in average HI± group scores wherein the HI+ patients scored less that the HI− in overall functional ability and sensory-motor components on the BI, FIM and RMI (Rivermead Mobility Index) [40], at discharge and up to one year post-stroke onset. Relatively less (statistically non-significant) disparity was found on cognitive FIM sub-scale scores in three studies [14,29,30]. Nevertheless, the magnitude of differences reported across studies was considerably variable even when the same measurement scales were used at discharge. Study [15] reported a difference between the HI± groups of 2 BI units (10%); whilst other studies reported differences of 7 BI units (35%) [31], 10 FIM units (8%) [14] and >30 FIM units (24%) [30]. However, it must be pointed out that time to 1st observation and discharge point were also considerably variable across the studies (see later section), therefore it is difficult to extrapolate further from the findings to isolate specific influences of HI.

### Progress rates

In general, similar rates of progress between the HI± groups were found prior to discharge but again these rates varied across studies even though the samples were homogenous with respect to lesion side (RHS). Four studies [10,14,27,31] followed up patients beyond discharge; study [27] found that specific HI training improved functional ability of the HI+ group but gains were not maintained by the end of the study (estimated from highly variable published data; recorded about 6–10 months after stroke).

### Length of in-patient stay and discharge destination outcome

The HI+ patient group tended to have longer LOS/days but this varied considerably across studies, e.g. HI+ 64, HI− 36 days [15], HI+ 31, HI− 25 days [12] and HI+ 79, HI− 52 days [31]. On average, levels of community support and rates of institutional care were higher in the HI+ patients. However, entry of both the groups to institutional care was variable; ranging from 1/40 (2.5%) [10], 32/178 (18%) [16] and 6/28 (22%) [31].

### Modelling results

In regard to multiple-regression modelling results, six out of eight studies reported that HI significantly and negatively predicted outcome in a variety of models of differing complexity and specification [10,16]. However, without a clear approach to modelling, such as identifying the strategy (e.g. step-up/down) used to build the models, the technique used to implement that strategy (forward/backward step-wise) and the decision criteria (e.g. theory driven) used within the technique, it was difficult to follow the modelling process and know the true effect of individual variables (inclusive of HI) as evident from the following examples.

Whilst four studies [10–12,16] reported independent prediction of HI for the measured functional outcomes, they did not clarify what “independent” means (e.g. explains more than 10% of the DV). In contrast, two studies [15,29] did not find a significant relationship between HI status and functional ability in any of the models evaluated – using either FIM or BI (0–20 scale) scores as the DV and HI as a categorical or continuous predictor variable, respectively. Notably the measurement of HI was itself a variable, since different studies used different measurement levels and scales.

In regard to covariates, pre-stroke function was unrelated to functional ability at admission (T0) according to three studies [15,16,30]. Baseline function was significantly, positively related to functional outcome at discharge according to three studies [12,28,29]. Age was significantly, negatively related to change in functional ability according to studies [28,29] but unrelated to final functional ability in models evaluated by studies [15,30]. Cognitive ability was unrelated to ADL as measured by BI and FIM in studies [15,30], respectively, but positively related in study [10].

### Overall model fit

HI generally explained little of the final variance in functional ability (DV). Study [30] reported the largest amount of variance explained in the DV (discharge FIM scores) as 49%; 44% of which were explained by admission FIM scores and a further 5% by BIT scores (HI levels). A key limitation of these analyses was that none of the studies undertook basic sensitivity analysis, e.g. how well the models met regression assumptions, such as normal distribution of residuals. In addition, confidence intervals (CIs) around regression coefficients or standard error (SE) sizes were rarely reported which complicated the interpretation of regression coefficients.

### Overall quality

Taking everything into account, individual studies were graded as C/D on the GRADE scale. This reflected (but was not limited to) serious limitations in the design and data analysis methods described in previous sections. In particular, the exclusion of RHS patients with higher levels of stroke severity and HI, effectively limited representativeness of the samples and generalisation to the wider clinical population. There was a lack of adjustment of potential confounding factors (such as stroke severity, age and time since stroke) in statistical models estimating specific effects of HI on outcomes; and insufficient data/details to enable the reader to make informed decisions (e.g. omission of confidence intervals around regression coefficient estimates which enable accurate interpretation of the result and lack of sensitivity analysis to support validity and conclusions made from the findings).

## Discussion

The purpose of the review was threefold – to test traditional claims that neglect/HI has a deleterious impact on functional ability after stroke, to assess the extent of differences between RHS patient groups (with and without HI) and clarify the relationship between HI and functional recovery. In addition, suggestions for more rigorous research were formulated based on a critique of these studies.

Based on findings from the 12 reviewed studies, it is apparent that the presence of HI+ is linked to poor functional outcomes in RHS patients when compared to their counterparts without HI impairments (HI−). However, it was not possible to assess the extent of differences between the HI± groups at specific points in time (elapsed after stroke) because of considerable variation and inconsistency in design within and across studies – not just in assessment measures but also in sample mix (age, stroke severity); time to 1st observation as well as follow-up assessments; and methods of data analysis. Neither was it possible to draw firm conclusions on the relationship between HI status and change in basic functional ability over time. In other words, the specific contribution of HI to the functional ability and outcome of RHS patients cannot be known with certainty from the results of the reviewed studies. It is possible that other influential factors, such as stroke severity, age and time since stroke substantially, explain the discrepancy in the HI± patient group scores reported in the literature. To this end, the implications of poor methodology in the field of stroke and HI were also highlighted in previous reports [2,21,22]. There is also recent discussion [38] of the importance of optimising data analysis methods in the field of neglect/HI to enhance the accuracy of predictive models.

Aside from clear methodological limitations, such as exclusion of patients with severe stroke and marked HI+ at baseline, it is likely that unmeasured patient factors also influenced the results and are responsible for a proportion of unexplained variance in functional ability post-stroke. These include pre-stroke educational levels and personality characteristics which would be expected to vary across patients. Furthermore, seven studies [12,15,16,28–30,32] used discharge as the only follow-up point. Although discharge is a key point of change in the stroke recovery pathway, it is subject to natural variation in stroke recovery rate and initial severity. Furthermore, the optimal discharge point may be influenced by differing cultural expectations as to when the recovering patient is ready to be discharged to community care and where this will be undertaken, i.e. home or institution. From the description available in past reviewed studies, it would appear that contextual features, such as stroke unit versus acute general rehabilitation hospital, were not sufficiently comparable in care and rehabilitation provision across different countries especially at the time that they were undertaken [10,12,14–16]. This lack of comparability in context of care is in itself a source of important variation when evaluating functional outcomes from different countries. In relation to the issues associated with discharge as an informative follow-up point, these can be minimised by including at least one standardised fixed follow-up point for all the patients, e.g. baseline – discharge – follow-up 6 months after stroke.

### Sample representation and size

Although all patients in the studies selected had RHS, there were considerable sample differences in age and recruitment settings which were not directly comparable. Furthermore, time to 1st observation and patient selection criteria were substantially different; and patients with severe cognitive impairment, and probably severe neglect/HI+ levels, tended to be automatically excluded at the recruitment phase. This strongly suggests that the samples were not sufficiently representative of the RHS population which limits the generalisation of any findings. It is imperative that future studies are conducted with representative (severity-inclusive) samples especially in predictive/explanatory relationship studies. This can be achieved by augmenting the design to suit specific requirements of severely cognitively impaired patients and then applying advanced methods of data analysis (e.g. multi-level modelling – MLM). Specific advantages of MLM over traditional methods of regression analysis and ANOVA are highlighted later on.

The importance of a large enough sample size in relation to study power and reducing type I error cannot be overemphasised. For instance, two studies [10,27] followed up patients for longer periods (up to one year after stroke) but with relatively small starting samples (40 and 59, respectively). Although attrition rate was not reported, this can be substantial at one year post-stroke which further reduces study power to detect differences between the HI± groups. Therefore, future studies should ensure sufficient initial sample size, particularly in longer term follow-up designs (lasting more than 3 months after stroke) to account for possible attrition; an allowance of 15–20% is cited in the literature [41,42].

### Measurement

The accuracy of results depended heavily on the precision of instruments and consistency in measurement. Only 50% of studies followed evidence-based recommendations in favour of diagnosing neglect/HI with validated test batteries rather than single cancellation task/s; the rationale [35,43,44] being that several tests are more likely to detect neglect than a single test. To this end, the proportion of the HI+ patients detected in studies that assessed with the BIT is considerably higher than in those that used single tests. Like other test batteries, the BIT has measurement limitations in that it is restricted to assessment of HI in activities conducted within close proximity (arm’s length) of surrounding body space and possibly mental representation of objects. In addition, similar to other test batteries (e.g. Chaterine Bergago scale [45]), the BIT cannot distinguish between different sub-types of the disorder [3,36] because all the assessed tasks inherently draw on visual, sensory, motor, spatial, perceptual and cognitive function to varying degrees. These components are intricately inter-twined and dependent on one another which make it virtually impossible to determine the relative contribution of individual components to the results or behaviour observed. Consequently (and of relevance to clinicians and researchers), it is not possible to accurately explain test results in terms of specific sub-types of HI in routine rehabilitation settings. That being said, assessment batteries provide an overall score of HI severity and are indicative of the type of perceptual, cognitive and/or motor impairments present.

Pending the development of more comprehensive and practical assessment tools, future researchers and clinicians should follow evidence-based assessment guidelines, i.e. use of validated assessment batteries for HI. Interpretation of results will also be enhanced by reporting the proportion of patients with mild compared to severe neglect/HI in the sample by categorising the HI+ patients at baseline. This is possible with the BIT [36,46].

Functional ability in the reviewed studies was assessed with at least two versions of the BI (using scales of 0–20 and 0–100) with differing sensitivity and precision [47,48], making comparison difficult. Furthermore, neither version assesses social/communicative components. They are therefore possibly more limited than the FIM in this respect [46]. In comparison, the Extended Barthel Index version has been used in several stroke studies [49,50]. It is a validated measure and overcomes some of the limitations of other BI versions in that it includes assessment of social and communicative components and accounts for time taken to complete ADL tasks.

Ideally future studies should measure functional ability with comprehensive evidence-based measures; however it must be acknowledged that current choices are limited not only by content in terms of validity but also reliability over time and applicability across acute and community settings.

### The position, interval and frequency of observations

In relation to design, the position, interval and frequency of observations is of critical importance in recording important changes in ability at the points when they are likely to occur. Furthermore, the amount and quality of information collected partly determines the type and extent of data analysis that is possible in order to answer specific research questions [39,51,52]. This ability was greatly compromised within and across the reviewed studies by the variability in time around the initial measurement point and follow-up, the number and frequency of observations made over time, and the duration of individual studies.

In some studies, the extent of variation around the mean LOS blurred the beginning and end of the study (e.g. study [16] reported mean LOS (HI+) 117 ± 61 days versus (HI−) 81 ± 38, and study [29] reported (HI+) 29 versus (HI−) 22, range 3–75 days). This would suggest considerable differences in LOS and patient exposure to care within and across studies which have implications for assessing recovery.

At baseline, the total variation in initial measurement points across studies was 7 days to 6 months post-stroke in studies [27,32], respectively. During this period, both spontaneous and rehabilitation-driven recovery processes are known to actively contribute to outcome [53–55]. Based on known recovery patterns, the impact of spontaneous recovery processes is expected to confound rehabilitative processes in at least 11/12 studies. Future studies should aim to collect initial data as early as is pragmatically possible, ideally within the 1st week of stroke. This would potentially streamline data collection procedures, enhance comparison of results across studies and minimise the effect of spontaneous recovery processes on outcome so that cause (e.g. influence of HI) can be more confidently inferred from regression results.

Future studies could also consider fixing the last follow-up point whilst still recording HI and functional change at discharge. Better still would be to statistically adjust for the confounding effect of time elapsed post-stroke. An optional growth curve modelling (MLM) approach could be used to model change over time in time-variant factors – this approach has been applied in a predictive stroke patient study [56]. The advantages afforded by MLM in relation to analysis of stroke data are next summarised in this review.

### Multi-level modelling approaches to data analysis

The advantages of multi-level modelling over traditional regression methods of analysis should not be overlooked when modelling dependency in the data due to multiple measurements from the same patient over time and interdependency between potential explanatory factors associated with functional ability (e.g. cognitive-motor processes). MLM can also handle data missing at random and unequal interval observations – all of which were potentially problematic features of the reviewed studies. As a result, MLM regression estimates are highly accurate and relatively stable compared to estimates obtained by ordinary multivariate regression analysis [51,52]. MLM has other useful features, such as estimation of unexplained variance within and across patients over time (in serial studies). It is imperative that future studies consider the advantages afforded by novel advanced statistical techniques in serial designs, which provide rich information but need to be appropriately statistically analysed for optimal results [38,57,58].

From the discussion so far it can be deduced that regression estimates in 8/12 studies which modelled outcome are likely to be overinflated with under-estimated standard errors. In turn, this increases the risk of type 1 error which has adverse implications for interpretation of results, not least the predictive importance of HI on functional ability.

### Confounding factors in stroke functional outcome studies

Another issue complicating the inference of causality from regression results is the lack of statistical adjustment for the established confounding effect of stroke severity and to a lesser extent chronological age. Besides the natural variation of both factors in different samples, stroke severity and age are both associated with HI [21,59,60]. Therefore, it is important that these particular variables are modelled in studies exploring multiple predictor variables and functional outcome [39,61]. As already stated, the use of MLM can greatly help with teasing out complicated relationship dynamics. By the same token, the need for an adequately powered study is emphasised. Considering that only six studies started with approximately 100 patients and some cases [10,27] also analysed predictive multivariate models, it is likely that respective studies were underpowered to detect true effects. This increases the uncertainty of the modelled results and detracts from the validity of their findings.

### Strengths and limitations of the review

The current review extended the work carried out by Jehkonen et al. by reviewing a homogeneous (versus mixed) patient sample with respect to hemispheric lesion (RHS). Further, a relatively more rigorous and systematic approach to study selection and the overall review process was undertaken.

The current review focused on group comparisons of functional outcomes of RHS patients with and without HI and the relationship of HI status with functional change with time since stroke, whereas Jehkonen et al. undertook a generalised review of the methodological issues from a wider range of studies which did not always include patient comparisons in the design (HI±). Group comparisons allow for the calculation of mean differences between patient groups and estimation of the modelled relationship between HI (group) status and functional outcome. This resulted in new insights into the data, such as the lack of adjustment for established confounding factors in past studies (e.g. stroke severity, time since stroke and age), leading to a different conclusion, i.e. no inferences could be made from the data available on either the relationship between neglect and functional outcome or the magnitude of difference in measureable scores between the patients with and without HI due to considerable heterogeneity in design and methods used to statistically analyse the data. Currently there is an urgent need for valid predictors and indicators to support the transfer of in-patient stroke rehabilitation services to appropriate stroke survivors living in the community.

Furthermore, due to the structure and layout of the review by Jehkonen et al., it is not possible to tell which studies found what and when in terms of results and time since stroke. The current review is more detailed in this respect. It is also more systematic and transparent both in the selection of studies and their evaluation (a checklist was used to ensure parity and consistency during the review process). The methodological quality of each study was separately graded. All these factors contribute to the rigour of the review and the validity of the findings.

The main limitation of the review is the possibility that relevant studies may have been missed due to indexing and multiple terms used to describe the “neglect” syndrome although a thorough search of the literature has been conducted several times. In addition, only English language publications or translations were considered.

The review does not address HI conditions arising from specific cortical or sub-cortical structures or anterior/posterior circulation division or left hemisphere lesions. Although HI is associated with different brain areas, HI emanating from the right hemisphere is commonly encountered in rehabilitation settings and challenging to treat [4–7,9]. In addition, the interpretation of results from assessments of HI in the left hemisphere lesions is confounded by speech and language impairments which frequently accompany LHS conditions. Non-language based clinical assessment for HI is urgently needed for this purpose. There is a lack of comparative group studies specifically on HI/neglect emanating from sub-cortical structures [63,64] probably because this is harder to identify in routine clinical settings and possibly less commonly encountered.

## Conclusions

To conclude, based on findings from this review, the evidence for an important relationship between HI status and functional outcome in RHS patients (with or without consideration of other influential factors) cannot be substantiated from the data available. This is due to significant methodological limitations of published studies highlighted in this review and the relatively few studies published in this field in the last decade [29–32]. The evidence that differences exist between the HI± patient groups on discharge and in the early months that follow tends to be better substantiated and is of interest to rehabilitation practitioners. However, on its own, this knowledge does not advance practice in the field.

In light of findings from this review, it is recommended that rehabilitation practitioners not only take into account the severity of HI in decision making but also recognise that evidence is weak for it being an independent marker of future functional recovery. Given the extent of differential progress possible in patients with RHS with and without HI, it is recommended that rehabilitation practitioners consider both the rate and amount of functional recovery over time. This is likely to be more informative overall than isolated measurements of HI soon after stroke. In relation to measurement, it is recommended that rehabilitation professionals sufficiently consider the psychometric limitations of commonly employed assessments of HI and overall functional ability (e.g. the BIT, BI and FIM) when interpreting the results.

Further research is warranted to identify the magnitude of the differences within and between the patient HI± groups and importantly the precise relationship between HI status and functional recovery with time since stroke. For this reason, well-designed, longitudinal, serial studies on large RHS patient samples are required which can withstand the scrutiny of future reviews on the subject.

It is recommended that future rehabilitation research in the field takes into account important methodological features to improve the quality and robustness of their findings. These include but are not limited to adequate sample size to detect differences in groups of patients with and without HI, with a full spectrum of stroke severity and HI, sufficient amounts of data by means of standardised methods repeated over time rather than isolated data points, and with sufficient follow-up to allow functional recovery to occur over time since stroke.

## Appendix 1.

**Table A1.** Critical evaluation checklist.

Internal and external validity	
1.	Is there definition of functional outcome and HI/Neglect?
2.	Is there a description of the design including setting/s, frequency of observations and time to first observation?
3.	Are the selection criteria clearly described?
4.	Has the stroke been confirmed (e.g. CT scan, MRI, neurological examination)
5.	Is the sample representative of the researched population?
6.	How has HI been identified and measured (test battery, single tests)
7.	Where other factors besides HI measured? If so how (measurement tool?)
8.	How was functional ability/outcome measured - is tool validated?
9.	What was the attrition rate – loss to follow-up & death?
Statistical validity	
10.	What was the sample size analysed (percentage of HI± patients known)?
11.	Where important confounding factors adjusted for (age, neurological severity, time)
12.	Type of statistical analysis undertaken?
13.	Do the results make sense? (Are they valid & useful?)
14.	Strength and limitations of study?

Abbreviations – CT = computer tomography, MRI = magnetic resonance imaging. Content was adapted from the Critical Appraisal Skills Programme [62].

## Appendix 2.

**Table A2.** Abbreviated terms.

1^a^	Primary
2nd	Secondary
ADL	Activities of daily living
ANOVA	Analysis of variance
BBS	Berg Balance Scale
BI	Barthel Index
BIT	Behaviour Inattention Test
CMSA	Chedoke-McMaster Impairment Inventory (measures neurological impairment
CNS	Canadian Stroke Scale
EMI	Elderly Mobility Scale
FIM	Functional Instrumental Measure
HI	Hemi-inattention
IADL	Instrumental activities of daily living
IV	Independent (predictor) variable
LOS	Length of stay
LOTCA	Lowenstein Occupational therapy cognitive assessment
MEAMS	Middlesex Assessment of Elderly Mental State
MMSE	Mini Mental State Examination
obs.	Observation
OT	Occupational therapy
PASS	Postural Assessment Scale For Stroke
PT	Physiotherapy
pt.	Patient
R^2^	Proportion of variance explained by a model
RCT	Randomised controlled trial
resp.	Respectively
RIC-FAS	Rehabilitation institute of Chicago functional assessment scale for comprehension and written expression
RKE	Rabideau Kitchen Evaluation
RMI	Rivermead Mobility Index
SD	Standard deviation
T0	Baseline
vs.	Versus
*β*	Regression coefficient
